# Selection on PER2 Phosphorylation Regulation Underlies Atypical Mammalian Circadian Rhythms

**DOI:** 10.1002/ece3.74524

**Published:** 2026-10-15

**Authors:** Yujie Chong, Qingqing Liu, Haojia Jiang, Linxue Tan, Huan Chen, Wenhua Ren, Shixia Xu, Guang Yang, Daiqing Yin

**Affiliations:** ^1^ Jiangsu Key Laboratory for Biodiversity and Biotechnology, College of Life Sciences Nanjing Normal University Nanjing Jiangsu China; ^2^ Southern Marine Science and Engineering Guangdong Laboratory (Guangzhou) Guangzhou Guangdong China

**Keywords:** atypical circadian rhythms, circadian plasticity, *CSNK1E* gene, PER2 phosphorylation, positive selection

## Abstract

Circadian rhythms are fundamental biological processes that enhance survival and reproductive fitness by coordinating physiology and behavior with predictable environmental cycles. However, in some species, circadian rhythms are weakened, irregular, or even undetectable, likely reflecting evolutionary responses to extreme environments or specialized ecological niches. Nevertheless, the genetic basis of these atypical phenotypes remains incompletely understood. Here, we investigated the molecular evolution of 20 PER2‐associated genes across 71 representative mammalian species. We identified four genes under positive selection in mammalian lineages exhibiting atypical circadian phenotypes, three of which (*CDK5*, *CSNK1E*, and *GSK3B*) are involved in the post‐translational regulation of PER2 phosphorylation, suggesting that PER2 phosphorylation regulators may contribute to lineage‐specific circadian variation. Notably, CSNK1E, a key kinase regulating PER2 phosphorylation, showed signatures of positive selection and accelerated evolution in cetaceans, which exhibit unihemispheric slow‐wave sleep (USWS). In vitro assays showed that dolphin CSNK1E increased phosphorylation at the stabilizing FASP site S662 of PER2 and extended the half‐life of the PER2 protein, a molecular feature closely linked to circadian oscillator amplitude and sleep regulation. Increased PER2 phosphorylation may contribute to altered sleep and locomotor activity associated with sustained wakefulness during USWS, as supported by our cellular and whole‐animal assays. Consistently, zebrafish overexpressing dolphin CSNK1E exhibited elevated locomotor activity and reduced sleep duration. Overall, our findings provide evidence that evolutionary changes in PER2 phosphorylation regulation are associated with atypical circadian phenotypes and offer novel insights into the genetic mechanisms underlying circadian plasticity in mammals.

## Graphical abstract

1. Positive selection on PER2 phosphorylation regulators in atypical circadian mammals. 2. Dolphin CSNK1E enhances PER2 phosphorylation at the stabilizing S662 site and extends PER2 half‐life. 3. Dolphin CSNK1E increases locomotor activity and reduces sleep in zebrafish.

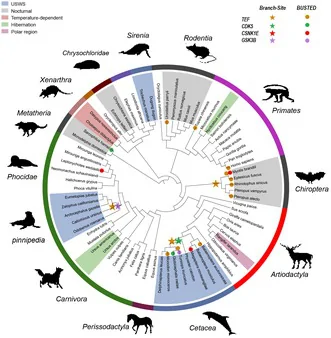

## 1 Introduction

Circadian rhythms are endogenous, approximately 24‐h cycles that occur in nearly all living organisms (Yamanaka 2020). They coordinate a broad range of physiological and biochemical processes, including the sleep–wake cycle, metabolism, and hormone secretion (Pan et al. 2020). By aligning internal timing with predictable daily environmental fluctuations, particularly daily light–dark cycles, circadian rhythms can reduce predation risk and facilitate reproductive success (Yerushalmi and Green 2009). Nevertheless, several mammalian lineages, including polar species, hibernators, nocturnal species, xenarthrans, and marine mammals, exhibit weakened, irregular, or even undetectable circadian behavioral rhythms, potentially reflecting lineage‐specific responses to extreme habitats or specialized lifestyles (Bloch et al. 2013; Li et al. 2022). For example, Arctic reindeer ( *Rangifer tarandus* ) remain active around the clock to exploit transient feeding opportunities during continuous summer daylight or winter darkness (Lin et al. 2019). In addition, marine mammals (except phocid seals) have evolved unihemispheric slow‐wave sleep (USWS), a distinctive sleep state associated with continuous movement and vigilance and characterized by partial divergence from canonical circadian regulation (Mascetti 2016). Understanding the genetic mechanisms underlying these atypical circadian phenotypes is essential for elucidating how circadian systems contribute to survival and reproduction under variable or extreme environmental conditions. However, the genetic basis of these atypical phenotypes remains largely unknown.

PER2 (Period circadian protein homolog 2), a core component of the negative feedback loop, helps maintain circadian rhythmicity by binding to CRY proteins to form a complex that translocates into the nucleus and inhibits CLOCK/BMAL1‐mediated transcription (Partch et al. 2014). Genetic studies have shown that PER2 deletion or disruptive mutations can lead to circadian disorganization or arrhythmicity, and human PER2 variants have been linked to familial advanced sleep phase syndrome (FASPS) (Toh et al. 2001; Xu et al. 2007). Comparative studies further suggest that lineage‐specific changes in clock components, including PER2, may play a role in atypical rhythms. For instance, species‐specific amino acid substitutions in PER2 have been reported in Arctic reindeer and the marine mammal dugong ( *Dugong dugong* ), with functional consequences for PER2‐CRY interactions that are proposed to support atypical circadian phenotypes (Li et al. 2022; Tian et al. 2024). Moreover, recent work in cetaceans indicates that cetacean‐specific mutations in BMAL2 can increase PER2 expression, potentially facilitating continuous movement and alertness during sleep (Yin, Zhang, et al. 2024). PER2 abundance and activity are regulated at both transcriptional and post‐translational levels. At the transcriptional level, D‐box‐binding transcription factors such as NFIL3 and DBP can compete for D‐box elements in the *PER2* promoter and modulate PER2 transcription. Functional evidence indicates that NFIL3 knockout lengthens the circadian period, while *DBP* overexpression shortens it (Mitsui et al. 2001; Yamajuku et al. 2011). Post‐translational regulation primarily involves phosphorylation as a key initiating process that controls the timing of subsequent modifications (Yamajuku et al. 2011; Chen et al. 2021). At the post‐translational level, kinases including CSNK1D, CSNK1E, GSK3B, and CDK5 dynamically phosphorylate PER2 to regulate its stability and subcellular localization, ultimately influencing circadian period length. The importance of PER2 phosphorylation is exemplified by the CSNK1E tau mutation, which produces a shortened circadian period and an advanced phase phenotype in both hamsters and mice (Militi et al. 2016; Narasimamurthy et al. 2018). In addition, ubiquitination and acetylation act together with phosphorylation to fine‐tune PER2 protein levels (Asher et al. 2008; Liu et al. 2018). Despite these advances, it remains unclear whether molecular changes in genes regulating PER2 are involved in the evolution of atypical circadian rhythms in mammals.

In this study, we focused specifically on the regulatory network controlling PER2, as this protein has been repeatedly linked to atypical circadian phenotypes (Li et al. 2022; Tian et al. 2024) and its phosphorylation is known to critically influence circadian period and sleep regulation (Zhou et al. 2015; Narasimamurthy et al. 2018). Accordingly, we conducted a comprehensive evolutionary analysis of 20 PER2 regulatory genes across 71 representative mammalian taxa. We found that genes involved in PER2 post‐translational phosphorylation showed an enrichment of positive selection signals in atypical rhythmic lineages. Subsequent cellular experiments showed that dolphin CSNK1E enhanced phosphorylation of PER2 at the stabilizing FASP site S662, extended the half‐life of PER2, and led to stronger repression of CLOCK/BMAL1‐mediated transcriptional activation. Furthermore, we conducted in vivo assays using zebrafish ( *Danio rerio* ), a vertebrate model widely used in sleep studies (Oikonomou and Prober 2017). This species exhibits diurnal sleep patterns characterized by established behavioral criteria and possesses conserved genetic and neurobiological features comparable to those of mammals (Miyazaki et al. 2017). Importantly, zebrafish provides several distinct advantages relative to traditional rodent models. It develops rapidly, exhibiting complex behaviors like sleep as early as 5 days post‐fertilization. Moreover, this species is highly amenable to high‐throughput behavioral testing and genetic modification (Oikonomou and Prober 2017). Although zebrafish peripheral clocks are directly entrainable by light, a feature distinguishing them from mammals, zebrafish have been widely used to study sleep regulation and circadian behavioral outputs, making zebrafish a valuable comparative platform for assessing the functional consequences of evolutionary changes in mammalian clock genes (Whitmore et al. 2000; Vatine et al. 2009). Our behavioral assays were therefore designed to evaluate conserved sleep–wake outputs. Using this system, we found that overexpression of dolphin CSNK1E significantly shortened sleep duration and increased sleep bout frequency, consistent with behavioral features involving reduced total sleep duration and enhanced vigilance observed in marine mammals exhibiting USWS. Collectively, our results support a model in which evolutionary modulation of PER2 phosphorylation, potentially affecting oscillator properties such as amplitude and stability, contributes to lineage‐specific circadian phenotypes, thereby advancing our understanding of the molecular and genetic basis of circadian plasticity in mammals.

## 2 Methods

### 2.1 Screening of Candidate PER2 Regulatory Genes

To investigate the molecular evolution pattern of genes involved in PER2 regulation, we established a curated set of candidate regulatory genes based on experimentally validated evidence from previous studies. Candidate genes were selected through a comprehensive review of the literature focusing on molecular mechanisms regulating PER2 expression, phosphorylation status, protein stability, and degradation. The inclusion criteria required that candidate genes have experimentally demonstrated roles in directly regulating PER2 transcriptional activity, mRNA abundance, post‐translational modification, protein stability, or degradation. A targeted literature search was conducted in PubMed using combinations of keywords, including “PER2 regulation”, “PER2 transcription”, “PER2 phosphorylation”, “PER2 stability”, “PER2 degradation”, “PER2 ubiquitination”, and “PER2 SUMOylation”. Published studies were manually evaluated to identify genes with experimentally supported roles in PER2 regulation. This process resulted in the identification of 20 candidate regulatory genes. These genes were further cross‐referenced with curated circadian pathway annotations from the KEGG (https://www.genome.jp/kegg/) circadian rhythm pathway (hsa04710) and the PER2‐associated protein interaction network available through STRING protein–protein interaction network (https://string‐db.org/network/9606.ENSP00000254657) to confirm their functional relevance within the circadian regulatory system. In addition, previously reported PER2 proteomic interactome data (Gul et al. 2022) were examined as additional supporting evidence for the functional relevance of the selected genes to PER2 regulation.

The 20 genes were classified into two functional groups according to their primary regulatory mechanism. The first group consisted of transcriptional and RNA‐level regulators involved in controlling PER2 expression, including NFIL3, DBP, HLF, TEF, CREB1, and PTBP1 (Yamajuku et al. 2011; Kim et al. 2020). The second group included post‐translational regulators that modulate PER2 protein stability, including BTRC, CDK5, CSNK1D, CSNK1E, GSK3B, MDM2, PPP1CA, PPP1CB, PPP1CC, PSMD11, SIK3, SIRT1, SUMO1, and SUMO2 (Reischl et al. 2007; Yamajuku et al. 2011; Cal‐Kayitmazbatir et al. 2023). Functional annotations, gene identifiers, and reported regulatory mechanisms were obtained from UniProt (https://www.uniprot.org/) and Ensembl databases (https://ensembl.org/) (Table S11).

### 2.2 Sequence Acquisition and Alignment

Coding sequences (CDSs) of the 20 PER2 regulatory genes were obtained from 71 phylogenetically diverse mammalian species, including 36 species representing canonical circadian patterns and 35 species exhibiting atypical circadian phenotypes (Table S12). Atypical species included taxa exhibiting USWS, such as cetaceans, sirenians, and amphibious pinnipeds, as well as polar‐dwelling reindeer, hibernators including pygmy lorises, black bears ( *Ursus americanus* ) and brown bear ( *Ursus arctos* ), and lineages described as temperature dependent in prior work (Bloch et al. 2013). Reference gene annotations and CDS sequences were collected from the National Center for Biotechnology Information (NCBI) database (https://www.ncbi.nlm.nih.gov/). Gene accession numbers were recorded for each species‐gene combination, and sequences were downloaded in batches using Batch Entrez (https://www.ncbi.nlm.nih.gov/sites/batchentrez). Gene accession numbers for each species–gene combination are provided in Table S12. To minimize potential errors caused by incomplete genome annotation, all retrieved sequences were subjected to additional quality control. Candidate CDSs were verified by BLASTn searches against the NCBI nucleotide database and by comparison with the corresponding reference protein sequences. Sequences containing premature stop codons, frameshift mutations, incomplete coding regions, or inconsistent coding sequence lengths were manually inspected and excluded or replaced when reliable alternative annotations were available. Orthologous sequences were identified based on Ensembl/NCBI gene annotations and further confirmed by sequence similarity and reciprocal BLAST analyzes to ensure that only one‐to‐one orthologs were retained for comparative evolutionary analyzes. All CDS sequences were translated into amino acid sequences to facilitate quality assessment and alignment validation.

Codon‐based multiple sequence alignments were generated using the PRANK algorithm implemented in FasParser2 (Sun 2018). The resulting alignments were manually inspected and adjusted using MEGA7 with MUSCLE implemented for additional alignment refinement (Kumar et al. 1994) to correct any obvious misalignments. Subsequently, Gblocks was applied with the parameter set “‐t = c” and “‐b5 = h” to remove hypervariable and poorly aligned regions (Castresana 2000). The resulting filtered codon alignments were used for subsequent evolutionary analyzes. The species topology was obtained from the time‐calibrated mammalian phylogeny available from TimeTree (http://www.timetree.org/) (Hedges et al. 2006) and the phylogenetic tree was formatted and visualized using iTOL (Letunic and Bork 2007).

### 2.3 Molecular Evolutionary Analyzes

Selective pressure acting on the 20 PER2 regulatory genes was evaluated by estimating the ratio of nonsynonymous substitutions (*d*_N_) to synonymous (*d*_S_) substitutions, represented as *ω* = *d*_N_/*d*_S_. Values of *ω* < 1 indicate purifying selection, *ω* = 1 represents neutral evolution, and *ω* > 1 suggest an excess of amino‐acid‐changing substitutions potentially consistent with positive selection. However, evidence for positive selection was inferred only when supported by likelihood‐based statistical tests and posterior probability analyzes. The *ω* values were estimated using the CODEML program in the PAML version 4.9e (Yang 2007) under a codon‐based maximum likelihood method. Atypical circadian lineages were additionally designated as foreground branches according to their ecological phenotype. Considering the potential biases from nucleotide substitution patterns and codon usage in molecular evolution, we used likelihood ratio tests (LRTs) to compare the nested models, with twice the difference in log‐likelihood values between alternative and null models calculated as the test statistic. To account for multiple comparisons among genes and evolutionary models, *p* values derived from LRT analyzes were corrected using the Benjamini‐Hochberg false discovery rate (FDR) method, with FDR < 0.05 considered statistically significant (Anisimova et al. 2001).

To identify genes undergoing accelerated evolution along specific lineages, we implemented the two‐ratio branch model in PAML. The null hypothesis (one‐ratio model) assumes that all branches share a single *ω* value, whereas the two‐ratio branch model allows foreground branches to have a distinct *ω* value from background branches. Major mammalian lineages, including Primates, Cetartiodactyla, Carnivora, Rodentia, Chiroptera, and other represented orders, were sequentially designated as foreground branches in independent analyzes, whereas all remaining branches were treated as background. Additionally, to detect lineage‐specific evolutionary shifts at finer phylogenetic resolution, each terminal branch was individually assigned as the foreground branch in separate analyzes. Branches showing significantly elevated *ω* values relative to background branches based on LRT comparisons were considered to exhibit lineage‐specific accelerated evolution.

To detect episodic positive selection affecting specific amino acid sites along particular mammalian lineages, we used the branch‐site model implemented in PAML to analyze the full mammalian dataset. For each analysis, a predefined lineage was designated as the foreground branch, while all remaining branches were treated as background branches. This model can detect positive selection at specific sites along a specific branch. Under the null hypothesis (Ma0), positive selection is not allowed, whereas under the alternative hypothesis (Ma), a class of sites on the foreground branches is allowed to have *ω* > 1. Under the null hypothesis (Ma0), sites are constrained to have *ω* values ≤ 1 across all branches, precluding positive selection. In contrast, the alternative hypothesis (Ma) permits a class of sites on the foreground branch to have *ω* > 1, indicating episodic positive selection. Positively selected sites were identified using the Bayes Empirical Bayes (BEB) approach, with sites showing posterior probabilities (PP) ≥ 0.8 (Yang and Nielsen 2002; Yang 2007).

To provide an independent assessment of episodic positive selection, we also applied the BUSTED (Branch‐Site Unrestricted Statistical Test for Episodic Diversification) model implemented in HyPhy, which provides an independent test for positive selection without relying on a predefined site‐class distribution (Kosakovsky Pond and Frost 2005; Kosakovsky Pond et al. 2020). Finally, to examine species‐specific patterns, we conducted branch specific analyzes in which each terminal branch was designated as the foreground branch in separate runs, enabling detection of lineage‐dependent selection signals at high resolution.

### 2.4 Detection of Convergent or Lineage‐Specific Amino Acid Substitutions

We used FasParser2 (Sun 2018) to screen for amino acid substitutions shared among two or more atypical lineages. Substitutions occurring in ≥ 80% of atypical lineages were considered candidate convergent substitutions. To test for convergence at deeper ancestral levels, we followed a phylogenetic framework described previously (Zou and Zhang 2015). Ancestral protein sequences were reconstructed using CODEML in PAML under an empirical amino acid model, and branch lengths were extracted from the inferred phylogenies. Site‐specific relative substitution rates were estimated using aaml in CODEML with the JTT matrix‐based method. Poisson statistics were then used to test for an excess of convergence events relative to random background substitutions, with adjusted *p* < 0.05.

To assess potential functional impact of convergent or lineage specific substitutions, we obtained domain annotations from UniProt and evaluated whether substitution sites fell within annotated functional domains. Human protein sequences were used as references for predicting functional consequences with PolyPhen‐2 (Adzhubei et al. 2010) and SIFT (Ng and Henikoff 2003). Variants were classified as deleterious if PolyPhen‐2 predicted them as possibly damaging or probably damaging, or if SIFT classified them as damaging. Three‐dimensional (3D) protein structures were predicted using AlphaFold v2.3.1 (Jumper et al. 2021) with default parameters. Protein–protein docking was performed with HDOCK. Predicted models were visualized in PyMOL (Version 4.6.0) to inspect the spatial position of substitution sites relative to functional domains. Docking poses were evaluated based on docking scores and ligand RMSD values. Due to the intrinsic limitations of rigid‐body docking and the presence of unstructured regions, the predicted orientations are considered exploratory and were not used to infer quantitative binding affinities.

### 2.5 Plasmid Construction

Total RNA was extracted from freshly isolated liver tissues of C57BL/6 mice using TRIzol Reagent (Invitrogen, Carlsbad, CA, USA; Cat. No. 15596018) according to the manufacturer's instructions. RNA concentration and purity were determined with a NanoDrop 2000 spectrophotometer (Thermo Fisher Scientific, Waltham, MA, USA). Samples with A260/A280 ratios between 1.8 and 2.0 were considered high‐quality. For cDNA synthesis, 1 μg total RNA was reverse transcribed using the PrimeScript RT Reagent Kit (Takara Bio, Shiga, Japan; Cat. No. RR047A) with oligo (dT) primers in a 20 μL reaction. The mouse *CSNK1E* open reading frame (ORF; NCBI accession no. NM_013767.8) was amplified from liver cDNA by PCR and cloned into the pIRES2‐EGFP vector (Clontech/Takara Bio) to generate an N‐terminal FLAG‐tagged construct with XhoI and ECORI sites. A synthetic FLAG‐tagged dolphin CSNK1E (NCBI accession no. XM_033866046.2) was obtained commercially (GenScript, Piscataway, NJ, USA). The mouse PER2 coding sequence (CDS) bearing an N‐terminal FLAG‐tag was synthesized by Tsingke Biotechnology (Beijing, China). These plasmids were used to transfect cells for subsequent western blot analysis. For zebrafish behavioral experiments, mouse and dolphin CSNK1E CDS were PCR amplified and cloned into the pCS2‐mCherry vector with BamH1 and ECORI sites. All constructs were verified by Sanger sequencing. PCR amplification was performed using the primers (Table S13).

### 2.6 Cell Culture and Transfection

HEK293T cells were obtained from our laboratory and were cultured in Dulbecco's Modified Eagle Medium (DMEM) supplemented with 1.8 mg/mL NaHCO₃, 4.5 mg/mL glucose, 100 U/mL penicillin, 100 μg/mL streptomycin, and 10% fetal bovine serum (FBS, SenBeiJia Biological Technology Co. Ltd., Nanjing, China). Cells were maintained at 37°C in a humidified incubator containing 5% CO₂. For transfection, cells were seeded in 6‐well or 12‐well plates 1 day before transfection and transfected to reach approximately 70%–80% confluency. Transfections were performed using jetPRIME reagent (Polyplus‐transfection, Illkirch, France, Cat. No. 101000046) according to the manufacturer's instructions.

### 2.7 Dual‐Luciferase Reporter Assay

Dual‐luciferase reporter assays were performed in 24‐well plates. HEK293T cells were transiently transfected with 525 ng total plasmid DNA per well, including 225 ng of a firefly luciferase reporter plasmid containing the target *PER2* or *PER1* promoter, 62.5 ng of mouse *CLOCK* expression plasmid, 62.5 ng of mouse *BMAL1* expression plasmid, 25 ng of Renilla luciferase control plasmid pRL‐SV40, 75 ng of mouse *PER2* expression plasmid, and 75 ng of the test plasmid such as mouse or dolphin *CSNK1E*. Transfections were carried out with jetPRIME reagent as described above. At 40 h post‐transfection, luciferase activities were measured using the Dual‐Luciferase Reporter Assay System (Vazyme Biotech Co. Ltd., Nanjing, China; Cat. No. DL101‐01). Firefly luciferase activity was measured first with a Synergy H1 Multimode microplate reader (BioTek, Winooski, VT, USA), followed by measurement of Renilla luciferase activity after addition of the Renilla substrate. Firefly activity was normalized to Renilla activity to account for variations in transfection efficiency, and results were reported as fold change relative to the appropriate control group. Each experiment was independently repeated at least three times, with three technical replicates per condition.

### 2.8 Protein Stability and Phosphorylation Assays

To evaluate the functional consequences of CSNK1E divergence on PER2 phosphorylation, we focused on the PER2 FASP site S662, a critical phosphorylation site involved in the PER2 phosphoswitch mechanism regulating PER2 stability (Narasimamurthy et al. 2018). To assess protein stability and phosphorylation, HEK293T cells were transfected with expression vectors encoding mouse CSNK1E‐Flag, dolphin CSNK1E‐Flag, mouse PER2‐Flag as indicated. Empty vector‐transfected cells were included as controls where appropriate. For protein degradation assays, at 36 h post transfection, cells were treated with cycloheximide (CHX; 200 μg/mL; MedChemExpress, Monmouth Junction, NJ, USA; Cat. No. HY‐12320) for 0, 3, 6, and 9 h to inhibit de novo protein synthesis. Cells were lysed in RIPA buffer (UnivBio, Shanghai, China; Cat. No. U10002) supplemented with 1 mM phenylmethylsulfonyl fluoride (PMSF; Sigma‐Aldrich, St. Louis, MO, USA; Cat. No. P7626) and protease/phosphatase inhibitor cocktail (Cat. No. 78440, Thermo Scientific). Lysates were mixed with SDS loading buffer (containing SDS, glycerol, bromophenol blue, and β‐mercaptoethanol) and heated at 95°C for 5 min. Equal amounts of protein (30 μg per lane) were separated by 8% or 10% sodium dodecyl sulfate‐polyacrylamide gel electrophoresis (SDS‐PAGE) and transferred to polyvinylidene difluoride (PVDF) membranes (0.45 μm; Merck Millipore, Billerica, MA, USA; Cat. No. IPVH07850). Membranes were blocked with 5% non‐fat milk in Tris‐buffered saline containing 0.05% Tween 20 (TBST) for 2 h at room temperature and incubated with primary antibodies overnight at 4°C. Primary antibodies included Mouse anti‐Flag (1:3000, ABclonal, Wuhan, China; Cat. No. AE005), Rabbit anti‐phospho‐PER2 (Ser662) (1:1000, Abcam, Cambridge, UK; Cat. No. ab206377), and Rabbit anti‐GAPDH (1:20,000, ABclonal, Wuhan, China; Cat. No. A19056) as a loading control. After washing with TBST, membranes were incubated with the appropriate HRP‐conjugated secondary antibodies for 1.5 h at room temperature. Protein bands were visualized using an enhanced chemiluminescence (ECL) kit (SuperFemto ECL Chemiluminescence Kit, Vazyme Biotech Co. Ltd., Nanjing, China; Cat. No. E423‐01) and imaged on a chemiluminescence imaging system. Relative PER2 protein abundance was quantified based on band intensity and normalized to the corresponding GAPDH signal. PER2 degradation kinetics were analyzed by fitting the relative protein abundance values at all indicated time points (0, 3, 6, and 9 h after CHX treatment) to a one‐phase exponential decay model in GraphPad Prism (version 8.0) using the equation: *Y* = (*Y*_0_ − Plateau) × exp. (−*K* × *X*) + Plateau, where *Y* represents the relative protein abundance at time *X*, *Y*_0_ represents the initial abundance (set to 100%), and *K* represents the degradation rate constant. The estimated protein half‐life was calculated as *t*₁/₂ = ln (2)/*K*. The goodness‐of‐fit of each decay curve was evaluated using *R* ^2^ values.

### 2.9 Construction of CSNK1E Overexpressing Zebrafish and Behavioral Analysis

Mouse and dolphin CSNK1E CDS were cloned into pCS2‐mCherry vector to generate fluorescent overexpression constructs. Larvae injected with pCS2‐mCherry empty vector served as injection control to account for any physiological effects of microinjection and exogenous mRNA overexpression. Plasmids were linearized with XbaI (New England Biolabs, Ipswich, MA, USA; Cat. No. R0145S) and purified by phenol‐chloroform extraction. Capped and polyadenylated mRNAs were synthesized using the mMESSAGE mMACHINE SP6 Transcription Kit (Thermo Fisher Scientific, Waltham, MA, USA; Cat. No. AM1340). For microinjection, mRNAs were diluted to 400 ng/μL in RNase‐free water, and approximately 2 to 3 nL was injected into one‐cell stage embryos of wild‐type AB strain zebrafish.

All zebrafish lines were housed on a 14 h/10 h light–dark schedule in dechlorinated water at 28.5°C and routine husbandry was performed by the zebrafish facility of Nanjing Normal University. Injected embryos were cultured in E3 medium containing 0.003% methylene blue at 28.5°C, and dead or deformed embryos were removed daily. At 72 h post fertilization (hpf), larvae displaying robust mCherry fluorescence were selected under a fluorescence stereomicroscope and placed individually into a 96‐well square bottom plate (Corning, NY, USA) containing 200 μL E3 medium per well. Behavioral monitoring was conducted under a 14 h light and 10 h dark cycle with lights on at 09:00 and lights off at 23:00. Locomotor activity and sleep parameters were recorded continuously for 72 h using a DanioVision Observation Chamber and analyzed using EthoVision XT version 14 (Noldus Information Technology, Wageningen, the Netherlands). Movement was tracked at 25 frames per second. Sleep was defined as a period of at least 1 min of continuous immobility, and sleep bouts were defined as discrete sleep episodes separated by wakefulness. All animal procedures were approved by the IACUC of the Animal Center, Nanjing Normal University (Approval No. IACUC‐20241105). The zebrafish behavioral assay system used in this study has been validated in our previous work (Yin, Yu, et al. 2024; Yin, Zhang, et al. 2024; Yin et al. 2025), in which control groups consistently exhibited modest but detectable day‐night differences under identical experimental conditions.

### 2.10 Statistical Analysis

All experiments were independently repeated at least three times using biological replicates, with *n* ≥ 3 per group unless otherwise stated. Statistical analyzes were conducted in GraphPad Prism 8 software (GraphPad Software, San Diego, CA, USA). Normality was evaluated using the D'Agostino and Pearson omnibus test and/or the Shapiro–Wilk test. For normally distributed data, one‐way ANOVA was applied with α = 0.05, followed by Holm–Šidák post hoc tests for pairwise comparisons. For non‐normally distributed data, the Kruskal–Wallis test was used, followed by Dunn's post hoc test with multiple‐comparison correction. Data are presented as mean ± standard error of the mean (SEM). Significance notation was indicated as follows: *p < 0.05, **p < 0.01, ***p < 0.001, ****p < 0.0001.

## 3 Results

### 3.1 Signatures of Accelerated Evolution and Positive Selection in Atypical Circadian Rhythmic Lineages

The branch model analyzes implemented in CODEML under the maximum likelihood framework of PAML (Yang 2007) identified signals of accelerated evolution in five genes (*CDK5*, *CSNK1E*, *GSK3B*, *NFIL3*, and *TEF*) along the terminal branches of 21 atypical circadian species, whereas only two canonical circadian species showed comparable signals after false discovery rate (FDR) adjustment (Table S1). Among these genes, four (*CDK5*, *CSNK1E*, *GSK3B*, and *TEF*) also exhibited accelerated evolution along 13 ancestral branches of atypical circadian mammalian lineages after FDR correction (Table S2). Specifically, the *CDK5* gene showed accelerated evolution only in USWS‐associated ancestral branches, the *CSNK1E* and *GSK3B* genes in both USWS‐associated and nocturnal ancestors, and the *TEF* gene in USWS‐associated, nocturnal, and temperature‐dependent xenarthran ancestors (Table S2).

The more stringent branch‐site model further identified signatures of positive selection in four genes (*CDK5*, *CSNK1E*, *GSK3B*, and *TEF*) along the terminal branches of 16 atypical circadian species, compared with only two canonical circadian species after FDR correction (Figure 1, Table S3). Three of these genes (*CDK5*, *CSNK1E*, and *TEF*) showed evidence of positive selection in both USWS‐associated and nocturnal lineages (Table S3), whereas the *GSK3B* gene was positively selected exclusively in USWS‐associated lineages (Figure 1; Table S3). To validate these findings with an independent framework, we applied the branch‐site unrestricted statistical test for episodic diversification (BUSTED) method implemented in HyPhy (Kosakovsky Pond and Frost 2005; Kosakovsky Pond et al. 2020). BUSTED provided additional evidence of positive selection in *CDK5*, *CSNK1E*, *GSK3B*, and *TEF* (Figure 1; Table S4). These four genes showed positive selection signals in the terminal branches of 18 atypical circadian species, compared with five canonical circadian species, indicating that positive selection was more frequently detected in atypical circadian lineages (Table S4). The lineage distribution was consistent with the CODEML results. *CDK5*, *CSNK1E*, and *TEF* showed positive selection on the terminal branches of USWS‐associated and nocturnal species, whereas positive selection signals in *GSK3B* were confined to USWS‐associated species (Figure 1, Table S4). In addition, three genes (*CDK5*, *GSK3B*, and *TEF*) showed positive selection along ancestral branches of USWS‐associated and nocturnal lineages, and the *TEF* gene also exhibited positive selection along the ancestral branch of chiropterans (Table S5).

**FIGURE 1:**
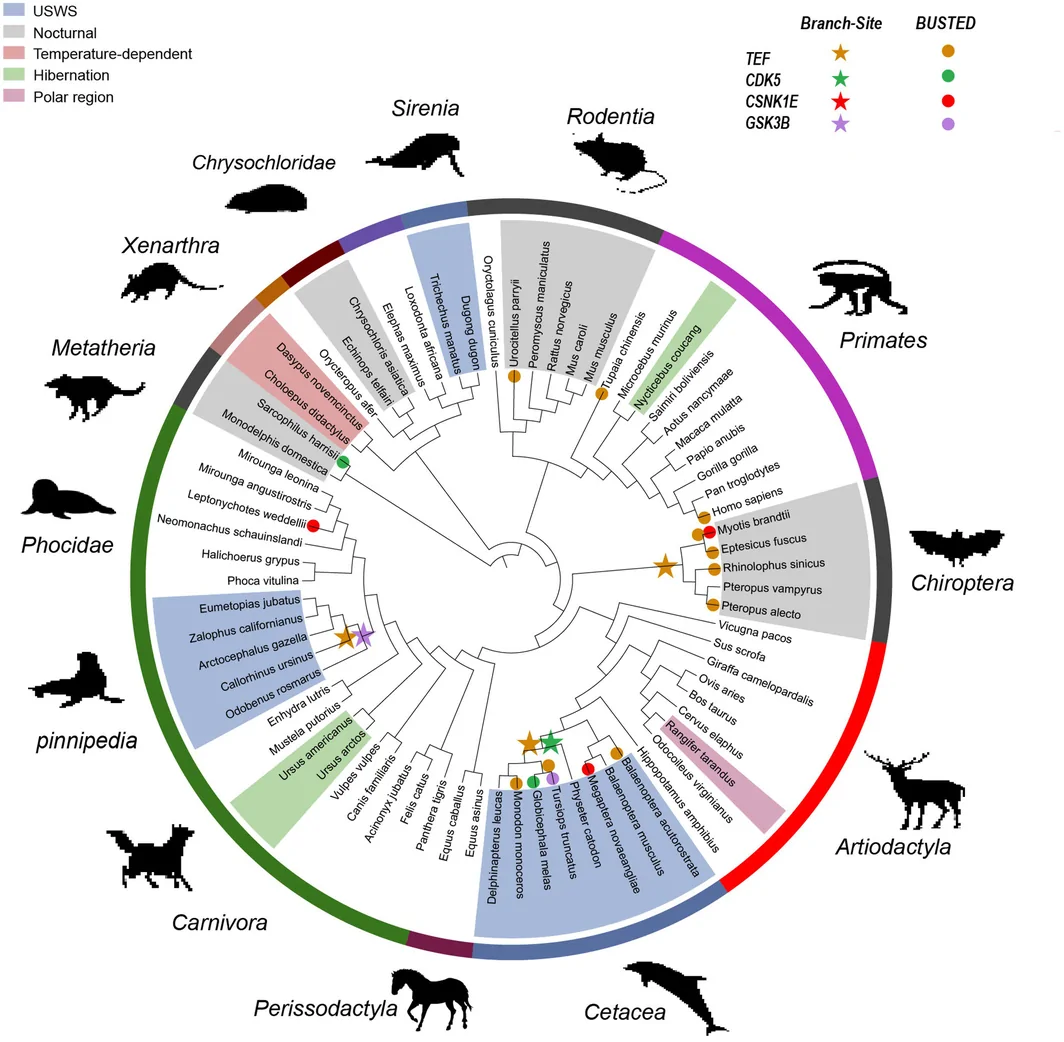
Time‐calibrated phylogeny of 71 mammalian species with atypical circadian lineages highlighted. A time‐calibrated phylogenetic tree of 71 mammals is shown, with lineages exhibiting atypical circadian phenotypes indicated by color. Categories include USWS (blue), nocturnal (gray), temperature‐dependent (pink), hibernating (green), and polar (purple), respectively. The tree was obtained from TimeTree and visualized in iTOL.

### 3.2 Lineage‐Specific Amino Acid Substitutions in Atypical Circadian Lineages

We used FasParser2 (Sun 2018) to screen for lineage‐specific amino acid substitutions in atypical circadian lineages that were absent from canonical circadian mammals. Overall, atypical circadian lineages contained approximately twice as many lineage‐specific amino acid substitutions in PER2‐regulated genes compared with canonical circadian lineages (Figure S1). Across 35 atypical circadian species, we identified a total of 60 lineage‐specific substitutions spanning 13 PER2‐regulated genes, whereas 36 canonical circadian species showed 29 such substitutions across 11 PER2‐regulated genes (Figure S1; Table S6). Several atypical circadian lineages displayed relatively high numbers of lineage‐specific substitutions. For example, 15 lineage‐specific substitutions were identified in cetaceans across seven genes, while no such substitutions were detected in their closely related ruminant lineages. Similarly, extensive lineage‐specific substitutions were observed in pygmy lorises (*Xanthonycticebus pygmaeus*) and reindeer, with 46 substitutions distributed across 16 PER2‐regulated genes in pygmy lorises and 32 substitutions across nine PER2‐regulated genes in reindeer, whereas no such substitutions were detected in their closely related primate and artiodactyl lineages, respectively (Table S7). Furthermore, FasParser2 did not identify any lineage‐specific substitutions that were shared among all atypical circadian lineages. Similarly, ancestral sequence reconstruction based on the JTT‐f_genes_ model (Zou and Zhang 2015) did not detect any convergent or parallel amino acid substitutions along these lineages.

### 3.3 Functional Domain Analysis of Lineage‐Specific Amino Acid Substitutions

Functional domain mapping showed that 55% of lineage‐specific amino acid substitutions in atypical circadian lineages, representing 33 of 60 sites, were located within or adjacent to annotated functional domains. In contrast, only 14 lineage‐specific substitutions in canonical circadian lineages fell within or near functional domains, including six in proboscideans and eight in perissodactyls (Figure S1; Table S6). Notably, pygmy lorises and reindeer harbored 27 and 11 substitutions within key domains, respectively (Table S7). Predictions using PolyPhen‐2 (Adzhubei et al. 2010) and SIFT (Ng and Henikoff 2003) algorithms indicated that 20 lineage‐specific substitutions in atypical circadian lineages were predicted to affect protein function, whereas only four potentially damaging substitutions were predicted in canonical circadian lineages (Table S6). Among canonical circadian lineages, proboscideans exhibited the largest number of lineage‐specific substitutions, with 16 total substitutions and three domain‐proximal variants predicted to affect protein function. By contrast, sirenians within the atypical group exhibited 18 substitutions, including eight located in key domains that were predicted to impact protein function. We further identified 34 lineage‐specific substitutions across four proteins, namely SIRT1, SIK3, NFIL3, and PTBP1. Notably, lineage‐specific substitutions in these four proteins were detected across all five atypical circadian lineages, although the affected residues and their domain distributions differed among lineages (Figures 2A and S2). We focused on substitutions located within or near annotated functional regions and predicted to have potentially deleterious effects on protein function. Within SIK3, four lineage‐specific substitutions (at positions 18, 28, 32, and 55) were located in the N‐terminal disordered region (residues 1–59), upstream of the protein kinase catalytic domain (residues 66‐317; UniProt Q9Y2K2), a region potentially involved in the regulation of SIK3 protein stability and function. In SIRT1, lineage‐specific substitutions were enriched in the CLOCK‐interacting region, suggesting a potential influence on CLOCK‐SIRT1 interaction and SIRT1‐mediated deacetylation of downstream targets (Figures 2B and S2). Notably, all three PER2 phosphorylation‐related genes showing evolutionary signals exhibited positive selection and accelerated evolution in USWS‐associated species. Further sequence analysis revealed that no specific sites were detected in CDK5, whereas GSK3B carried four cetacean‐specific substitutions located in a low‐complexity region. Functional predictions and structural analyzes indicated that these mutations do not significantly affect protein structure or function (Table S8). In contrast, CSNK1E, a key kinase that directly phosphorylates PER2 protein (Kloss et al. 1998; Keesler et al. 2000), carried five cetacean‐specific substitutions in its C‐terminal tail (Figure 3A), which is implicated in the regulation of phosphorylation. Molecular docking results further provided preliminary structural insights into the interaction between CSNK1E and PER2. The predicted docking models suggested a trend toward reduced hydrogen bond interactions between dolphin CSNK1E C‐terminal region and PER2 compared with the mouse ortholog, although individual substitutions had limited effects on the three‐dimensional structure of CSNK1E (Figure 2C,D; Tables S9 and S10).

**FIGURE 2:**
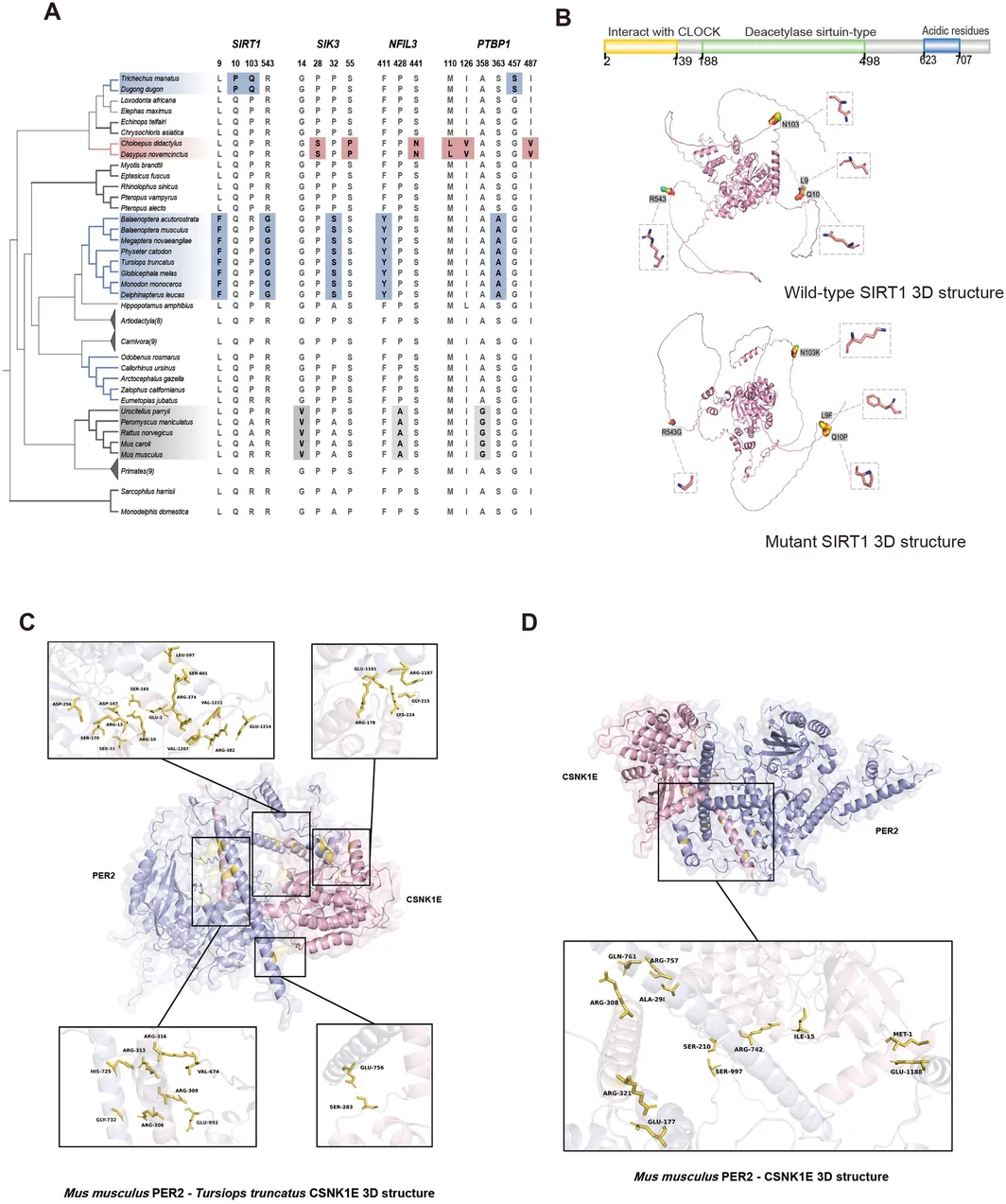
Structural features and sequence conservation of PER2‐regulated genes. (A) Multiple sequence alignments highlight representative lineage‐specific substitutions in key PER2 regulatory genes. Substitution sites are indicated below the alignments, and species belonging to USWS (blue), nocturnal (gray), and temperature‐dependent (red) categories are marked. (B) Structural mapping of four radical substitutions predicted to be damaging in SIRT1. (C) Docking complexes of mouse PER2 (purple) with mouse CSNK1E (pink). (D) Docking complexes of mouse PER2 (purple) with dolphin CSNK1E (pink). The docking poses generated using HDOCK. Visualizations were prepared in PyMOL.

**FIGURE 3:**
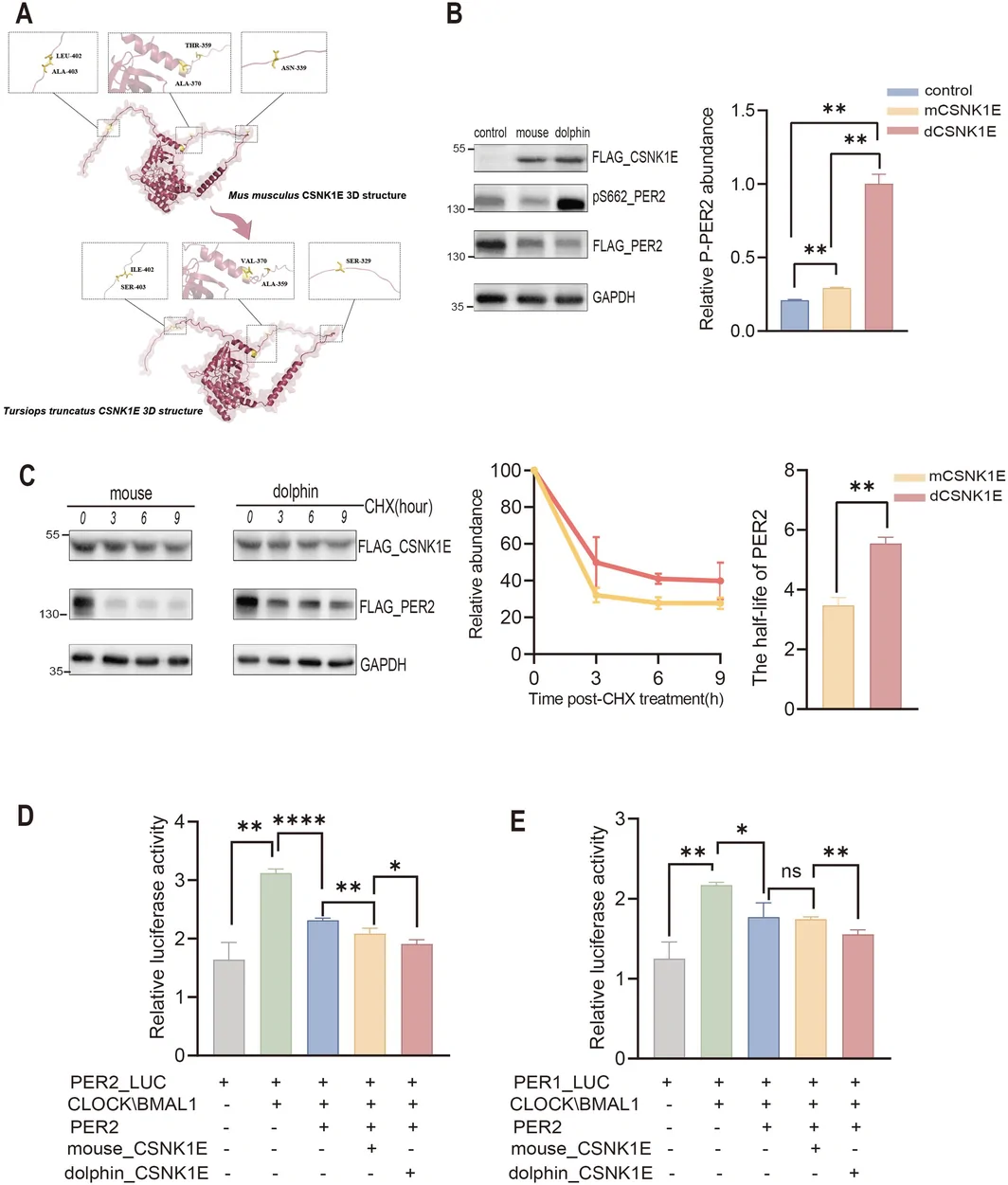
In vitro functional assays show that dolphin CSNK1E increases PER2 phosphorylation and stabilizes PER2 proteins. (A) Three‐dimensional structures of CSNK1E from mouse ( *Mus musculus* ) and bottlenose dolphin ( *Tursiops truncatus* ) were predicted using AlphaFold and visualized in PyMOL. Cartoon representations are colored by secondary structure (or as shown). (B) Western blot analysis and quantification of PER2 phosphorylation Ser662 in HEK293T cells co‐expressing FLAG‐PER2 with either FLAG‐mouse CSNK1E (yellow bars) or FLAG‐dolphin CSNK1E (red bars). Protein levels were detected using anti‐pSer662‐PER2 antibody (mean ± SEM) from three independent experiments. (C) Western blots show the protein stability assays of N‐terminal FLAG‐tagged mouse PER2 co‐expressed with FLAG‐tagged mouse or dolphin CSNK1E in HEK293T cells following CHX treatment at 200 μg/mL. GAPDH served as a loading control. Half‐life was determined by using nonlinear, one‐phase exponential decay analysis (half‐life parameter, K, is significantly different among all two proteins: *p* < 0.01). Quantified half‐lives are shown below each blot (mean ± SEM) from three independent experiments. (D, E) Dual‐luciferase reporter assays assess the effects of mouse or dolphin CSNK1E on CLOCK and BMAL1‐driven activation of *PER2* (D) and *PER1* (E) promoter reporters in HEK293T cells (mean ± SEM) from three to four independent experiments. Statistical significance: ****p < 0.0001, ***p < 0.001, **p < 0.01, *p < 0.05.

### 3.4 In Vitro and In Vivo Validation of Dolphin CSNK1E Effects on PER2 Phosphorylation and Sleep‐Related Phenotypes

To investigate the functional impact of the identified lineage‐specific evolutionary changes in atypical circadian mammals, we focused on CSNK1E (casein kinase I isoform epsilon), which is a key circadian regulator, modulates phosphorylation of the core clock protein PER2, and contributes to the determination of circadian period length (Etchegaray et al. 2009; Narasimamurthy et al. 2018). Notably, our evolutionary analysis revealed that CSNK1E shows accelerated evolution and positive selection in cetaceans, a lineage characterized by unihemispheric slow‐wave sleep (USWS), a sleep phenotype decoupled from canonical circadian regulation. These findings identify CSNK1E as a candidate gene for further functional investigation of the molecular mechanisms underlying atypical rhythm phenotypes. The S662 site has been reported to act as a switch for initiating PER2 phosphorylation and is critical for regulating PER2 stability (Narasimamurthy et al. 2018). Therefore, we first compared the effects of mouse and dolphin CSNK1E on PER2 phosphorylation in HEK293T cells using an antibody specific for phosphorylated S662. PER2 phosphorylation was significantly higher in cells expressing dolphin CSNK1E than in cells expressing mouse CSNK1E (*p* = 0.0041) (Figure 3B). Since PER2 phosphorylation is closely linked to protein stability, we next assessed PER2 turnover after cycloheximide (CHX) treatment to inhibit de novo protein synthesis. Based on the observed degradation kinetics, PER2 showed an estimated half‐life of 5.55 ± 0.25 h in dolphin *CSNK1E*‐transfected cells, approximately 60% longer than that estimated in mouse *CSNK1E*‐transfected cells (3.48 ± 0.2 h; *p* < 0.01) (Figures 3C and S4). Together, these results indicate that dolphin CSNK1E enhances PER2 phosphorylation and is associated with increased PER2 stability. We then assessed transcriptional output using dual‐luciferase reporter assays. Specifically, in CLOCK/BMAL1‐driven *PER2*‐ or *PER1*‐promoter luciferase assays, cells were co‐transfected with PER2; dolphin CSNK1E reduced reporter activity more strongly than mouse *CSNK1E* (the *PER2* promoter, *p* = 0.0368; the *PER1* promoter, *p* = 0.0082; Figure 3D,E), in line with the increased PER2 stability conferred by dolphin CSNK1E.

To examine organismal consequences, we monitored locomotor rhythms in zebrafish larvae overexpressing dolphin CSNK1E, mouse CSNK1E, or an EGFP‐negative control under a light–dark (LD) cycle for 3 days using an automated tracking system. All groups maintained robust circadian rhythmicity (Figure 4A). Larvae overexpressing dolphin CSNK1E exhibited significantly greater total locomotor distances than larvae overexpressing mouse CSNK1E (1111.0 ± 112.9 cm vs. 840.7 ± 56.08 cm; *p* = 0.0354) (Figure 4B). The difference was most pronounced during the light phase (695.6 ± 71.74 cm vs. 481.6 ± 41.79 cm; *p* = 0.0143) and remained evident on subsequent days, including day 6 (299.4 ± 48.58 cm vs. 157.4 ± 15.22 cm; *p* = 0.0070) and day 7 (253.1 ± 18.39 cm vs. 143.2 ± 19.9 cm; *p* < 0.001) (Figure 4C). Sleep analyzes further showed that dolphin CSNK1E overexpression reduced total sleep time relative to both the mouse CSNK1E group and EGFP‐negative controls. Total sleep time was 2456 ± 247.4 min in dolphin CSNK1E group versus 3475 ± 88.86 min in the mouse CSNK1E group (*p* = 0.0006) and 3239 ± 100.5 min in controls (*p* = 0.0293) (Figure 4D). During the dark phase, larvae overexpressing dolphin CSNK1E slept less than those overexpressing mouse CSNK1E (1555 ± 23.36 min vs. 1632 ± 11.64 min; *p* = 0.0049), consistent with reduced sleep on night 5 (509.7 ± 11.46 min vs. 535.8 ± 5.75 min; *p* = 0.0451) (Figure 4E). Sleep architecture indicated that reduced sleep in dolphin CSNK1E group was driven mainly by shorter sleep bout length during the dark phase (*p* = 0.0461) (Figure 4F) and an increased number of sleep bouts during the light phase (*p* = 0.0473) (Figure 4G), rather than differences in sleep latency at the light–dark transitions (Figure S3). Collectively, these data show that overexpression of dolphin CSNK1E alters locomotor activity and sleep‐related behavioral outputs in zebrafish.

**FIGURE 4:**
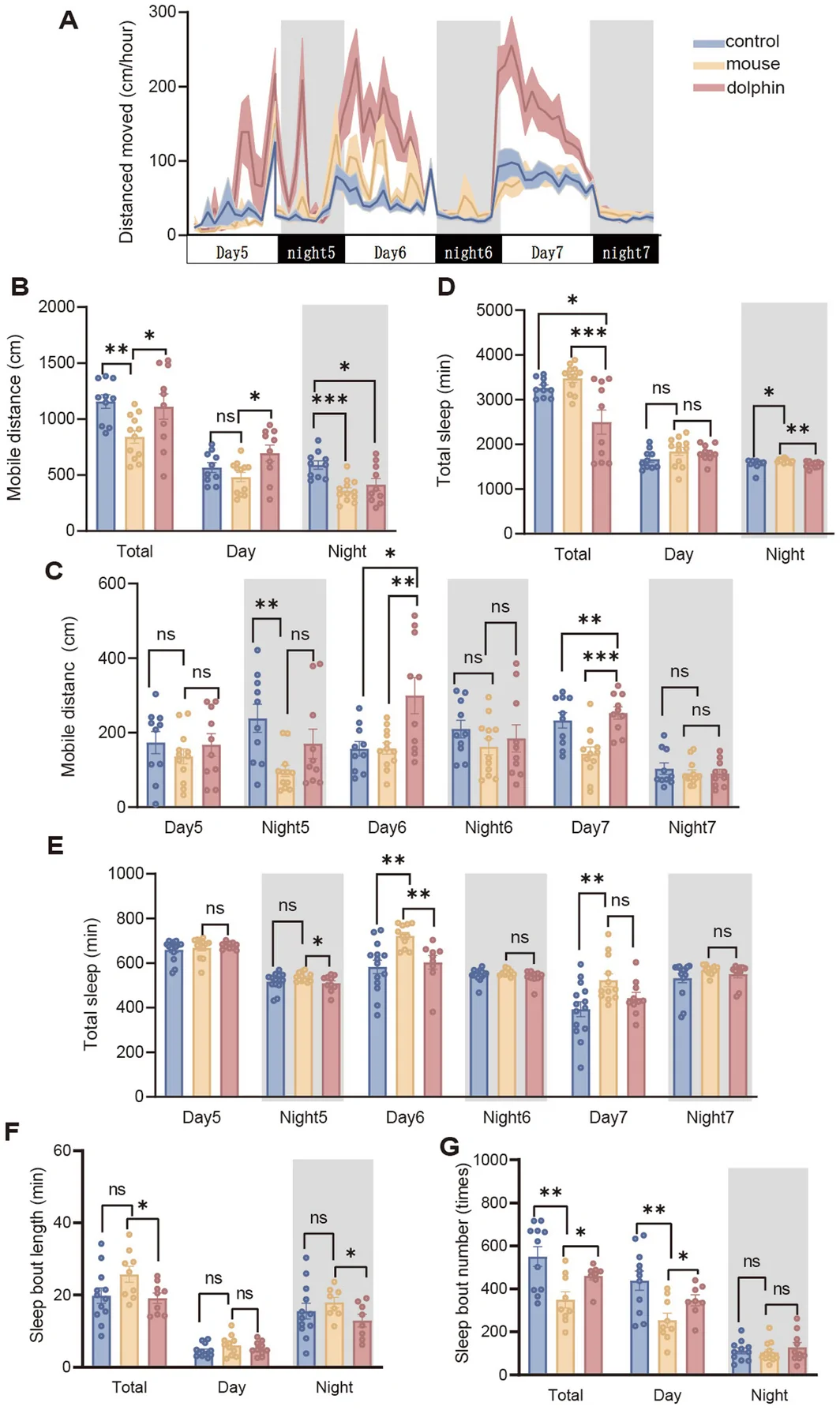
Dolphin CSNK1E overexpression alters locomotor activity and sleep in zebrafish larvae. (A) Representative 72 h locomotor activity traces are shown for zebrafish larvae overexpressing pcs2‐mCherry vector as a control (*n* = 10), mouse CSNK1E (*n* = 12), or dolphin CSNK1E (*n* = 10), all behavioral assays were performed under a 14:10 LD cycle. (B, C) Total movement distance measured across 3 days (B) and partitioned by light and dark phases (C) (mean ± SEM). (D, E) Total sleep measured across 3 days (D) and partitioned by light and dark phases (E) (mean ± SEM). (F) Average sleep bout length during the dark phase. (G) Number of sleep bouts during the light phase. Statistical significance: ****p < 0.0001, ***p < 0.001, **p < 0.01, *p < 0.05.

## 4 Discussion

Circadian rhythms are evolutionarily conserved endogenous timekeeping mechanisms that oscillate with an approximately 24‐h period and are present across most living organisms (Bell‐Pedersen et al. 2005). They coordinate diverse physiological and biochemical processes, enabling organisms to anticipate predictable daily environmental fluctuations (Patton and Hastings 2023). By aligning behavior and physiology with external environmental cycles, circadian systems can enhance survival and reproductive success while facilitating adaptation to specific ecological niches. Although most species exhibit canonical near 24‐h rhythms, some lineages have evolved strategies that partially decouple locomotor activity from strict circadian regulation and instead synchronize behavior with specific environmental cues (Bloch et al. 2013). However, the molecular mechanisms underlying these evolutionary shifts in mammals with atypical circadian phenotypes remain largely unresolved.

Previous studies investigating atypical circadian rhythms have primarily focused on individual species and have emphasized potential evolutionary links between PER2 and atypical rhythmic phenotypes (Li et al. 2022; Tian et al. 2024). In the present study, we expanded this perspective by performing a comparative evolutionary analysis of 20 PER2‐regulated genes across 71 representative mammalian taxa. Using CODEML‐based analyzes implemented in PAML, we detected lineage‐specific evolutionary changes in 18 of the 20 examined genes across atypical circadian lineages. Notably, 39% of these genes, representing 7 of 18, were shared across three distinct atypical lineages (Figure 1). Within this broader evolutionary landscape, four genes (*TEF*, *CDK5*, *CSNK1E*, and *GSK3B*) showed strong signatures of positive selection in atypical circadian lineages. Among these genes, *TEF* exhibited positive selection signals in both USWS‐specific and nocturnal species. TEF functions as a direct and rapid light‐responsive transcription factor in early vertebrates and subsequently serves as a key light‐activated molecule in mammals (Vatine et al. 2009). Nocturnal and marine mammals all inhabit relatively low‐light environments, including nocturnal environments and deep aquatic habitats, and often exhibit enhanced sensitivity to dim light conditions (Cobo et al. 2025). Therefore, lineage‐specific evolutionary changes in *TEF* may influence light perception or downstream responses in these specialized ecological contexts. Furthermore, *CDK5*, *CSNK1E*, and *GSK3B* are known regulators of PER2 post‐translational phosphorylation (Iitaka et al. 2005; Militi et al. 2016; Brenna et al. 2019). Across these three genes, we identified 176 positively selected sites, of which 72% were located within conserved protein kinase domains that are essential for phosphorylation activity (Johnson 2001). Phosphorylation represents one of the prominent post‐translational modifications regulating core clock components and is crucial for circadian timing across diverse taxa (Robles et al. 2017; Brüning et al. 2019). Compared with other regulatory modifications, such as acetylation and ubiquitination, phosphorylation can directly modulate protein activity through kinase‐mediated reactions, enabling rapid signal transduction and dynamic regulation of cellular processes (Patwardhan and Miller 2007; Kleiger and Mayor 2014; Yu et al. 2025). Therefore, the enrichment of evolutionary signals in PER2 phosphorylation regulators suggests that these genes may represent important candidate genes associated with altered phosphorylation regulation in mammals exhibiting atypical circadian phenotypes.

The functional relevance of PER2 phosphorylation‐related genes is further supported by our cellular and whole animal assays. CSNK1E is one of the first kinases identified to directly phosphorylate PER2 in mammals and plays a role in regulating circadian period length through PER2 phosphorylation (Kloss et al. 1998; Narasimamurthy et al. 2018). Specifically, CSNK1E directly phosphorylates the PER2 phosphorylation switch site S662, which primes PER2 for subsequent phosphorylation events, while the C‐terminal region of CSNK1E has been implicated in regulating kinase activity and circadian rhythmicity. Our molecular docking analyzes suggest that, compared with mouse CSNK1E, dolphin CSNK1E carries five cetacean‐specific substitutions within the C‐terminal region, which may influence hydrogen bonding interactions with PER2 and potentially alter the interaction mode. The C‐terminal tail of CSNK1E is intrinsically disordered and undergoes autophosphorylation that negatively regulates kinase activity through the pseudosubstrate mechanism (Gietzen and Virshup 1999; Cullati et al. 2024; Harold et al. 2024). Given that these cetacean‐specific substitutions are located within this regulatory tail rather than the conserved catalytic domain, they may modulate CSNK1E activity by affecting autoinhibitory regulation. This provides a potential mechanistic explanation for the increased PER2 phosphorylation observed in cells expressing dolphin CSNK1E, despite the absence of detectable structural changes in the folded kinase domain. Subsequently, cellular assays using a pS662‐PER2‐specific antibody showed that dolphin CSNK1E increased PER2 phosphorylation compared with mouse CSNK1E. Prior studies have established the “PER2 Phosphoswitch” model, in which phosphorylation at FASP priming sites (S662) promotes PER2 stabilization (Zhou et al. 2015; Narasimamurthy et al. 2018; Masuda et al. 2020). In line with this, our protein stability experiments also demonstrated that dolphin CSNK1E can stabilize PER2 and prolong PER2 protein half‐life. However, CSNK1E is not restricted to PER2 and can also phosphorylate other circadian regulators, including BMAL1, CRY proteins, and E4BP4/NFIL3 (Eide et al. 2002; Doi et al. 2004). Thus, although our results support altered PER2 phosphorylation and stability as a potential mechanism associated with the observed phenotypes, other CSNK1E‐dependent pathways may also contribute, and the present data do not establish PER2 phosphorylation as the sole causal mechanism. Nevertheless, PER2 represents a biologically plausible downstream pathway because it plays a crucial role in regulating circadian rhythms and sleep‐related processes. Theoretical and experimental studies indicate that appropriate PER2 degradation kinetics are required for maintaining high‐amplitude circadian oscillations and consolidated wake–sleep cycles (D'Alessandro et al. 2017). Computational modeling further suggests that reduced PER2 degradation rates can decrease oscillator amplitude under specific conditions (Kim and Forger 2012). Consistently, PER2‐null mutant zebrafish show decreased activity and delayed rhythm phases, whereas PER2 knockout mice exhibit disrupted activity rhythms under constant darkness (Wang et al. 2015; Riggle et al. 2022). In contrast, increased PER2 expression levels have been associated with reduced sleep duration and impaired sleep recovery following sleep deprivation in mice (Franken et al. 2007; Millius et al. 2023). In addition, CSNK1E mutations are closely related to emotional and behavioral changes under rhythm disorders, such as fragmented sleep and reduced rhythm amplitude (Kripke et al. 2009; Möller‐Levet et al. 2013). Furthermore, CSNK1E is involved in regulating REM sleep amount and NREM sleep fragmentation. Specifically, CSNK1E mutant mice exhibit a higher proportion of REM sleep during the dark phase, whereas total sleep and circadian period length do not show any significant changes (Zhou et al. 2014), suggesting that the role of CSNK1E in regulating sleep is likely independent of the circadian rhythm. Therefore, these findings suggest that the functional consequences of dolphin CSNK1E evolution may involve multiple aspects of sleep and circadian regulation. First, increased CSNK1E‐mediated PER2 phosphorylation may affect the amplitude of circadian rhythm and circadian feedback process, which may contribute to the evolution of a unique sleep pattern that is less constrained by circadian control and allows for sleep–wake transitions and flexible alteration of sleep between the two hemispheres. Theoretical and molecular studies suggest that the evolutionary basis of USWS involves partial decoupling of sleep–wake regulation from conventional circadian control, allowing sustained movement and vigilance (Kedziora et al. 2012; Yin, Yu, et al. 2024; Yin et al. 2025), as their movement is nearly continuous throughout life to prevent drowning and maintain thermogenesis (Lyamin et al. 2005; Siegel 2008; Kedziora et al. 2012; Yin, Yu, et al. 2024; Yin et al. 2025). Second, altered CSNK1E activity and the consequent changes in PER2 expression are beneficial to behavioral states characterized by enhanced locomotor activity and reduced sleep propensity. Our previous study also found that two cetacean‐specific BMAL2 substitutions can enhance the expression of PER2 and wake‐promoting substances, which facilitates sustained wakefulness and locomotion during sleep (Yin, Zhang, et al. 2024). Consistent with these findings, zebrafish larvae overexpressing dolphin CSNK1E showed reduced total sleep duration and an increased number of sleep bouts compared with larvae overexpressing mouse CSNK1E (Figure 4D,G). We acknowledge that zebrafish and mammalian circadian systems differ in their regulatory architecture, particularly because zebrafish peripheral clocks are directly responsive to light. Therefore, this heterologous assay does not fully recapitulate mammalian circadian regulation. In addition, control zebrafish did not show statistically significant day‐night differences under our experimental conditions, which may reflect behavioral variability and limitations associated with transient overexpression systems. Future studies using stable transgenic zebrafish lines and longer recording periods will help further characterize the functional effects of CSNK1E variants. Nevertheless, comparisons between mouse and dolphin CSNK1E under identical experimental conditions provide evidence that these orthologs have divergent effects on sleep‐related behavioral outputs. Collectively, our findings support the hypothesis that lineage‐specific molecular modifications in circadian regulators may contribute to the evolution of specialized sleep phenotypes in cetaceans.

## 5 Conclusions

In this study, we investigated the molecular evolutionary mechanisms underlying atypical circadian phenotypes in mammals. We identified strong signals of positive selection in atypical lineages, particularly among genes involved in PER2 phosphorylation regulation. At the cellular level, we focused on the PER2 priming phosphorylation site S662 and demonstrated that dolphin CSNK1E increases phosphorylation at this stabilizing FASP site, extends PER2 half‐life, and strengthens repression of CLOCK/BMAL1‐mediated transcriptional activation. These molecular changes may contribute to altered circadian feedback regulation and sleep‐related behavioral adaptations associated with atypical circadian phenotypes. This is further supported by the observation that zebrafish overexpressing dolphin CSNK1E exhibited increased locomotor activity and shortened sleep duration. Together, these results indicate that positive selection on PER2 phosphorylation regulators, together with functional validation of CSNK1E, provides a link between lineage‐specific molecular evolution and altered PER2 phosphorylation, clock transcriptional output, and sleep‐related traits, thus potentially contributing to atypical circadian phenotypes in specialized ecological contexts. Overall, our findings advance our understanding of the evolutionary forces shaping circadian diversity and provide mechanistic insights into how genetic changes may contribute to circadian plasticity in mammals. However, the present approach had several limitations, including variability in co‐transfection efficiency among individual expression constructs, which cannot be fully corrected by Renilla normalization. Additional studies using direct quantification of endogenous gene expression and complementary approaches will further clarify the regulatory effects of dolphin CSNK1E.

## Author Contributions

**Yujie Chong:** conceptualization (equal), formal analysis (equal), project administration (equal), visualization (equal), writing – original draft (equal), writing – review and editing (equal). **Qingqing Liu:** data curation (equal), investigation (equal). **Haojia Jiang:** data curation (equal). **Linxue Tan:** investigation (equal). **Huan Chen:** investigation (equal). **Wenhua Ren:** data curation (equal), investigation (equal), project administration (equal). **Shixia Xu:** data curation (equal), investigation (equal), project administration (equal). **Guang Yang:** conceptualization (equal), funding acquisition (equal), writing – review and editing (equal). **Daiqing Yin:** conceptualization (equal), funding acquisition (equal), supervision (equal), writing – review and editing (equal).

## Funding

This work was supported by the National Natural Science Foundation of China (Grants 32030011, 32200348 and U24A20362), the PI Project of Southern Marine Science and Engineering Guangdong Laboratory (Guangzhou) (Grant GML2021GD0805), the Priority Academic Program Development of Jiangsu Higher Education Institutions (PAPD), and the Postgraduate Research & Practice Innovation Program of Jiangsu Province (Grant KYCX24_1854).

## Conflicts of Interest

The authors declare no conflicts of interest.

## Supporting information


**Data S1:** Supporting Information.

**Figure S1:** Distribution of lineage‐specific amino acid substitutions in PER2 regulatory genes across mammalian circadian phenotype groups. Horizontal bar plots show the number of PER2 regulatory genes containing lineage‐specific amino acid substitutions and the total number of lineage‐specific amino acid substitutions identified in each circadian phenotype group. Atypical circadian groups include USWS‐associated, nocturnal, temperature‐dependent, hibernating, and polar lineages, with canonical circadian mammals shown for comparison. Numbers within or adjacent to the bars indicate the corresponding counts of affected genes and amino acid substitutions.
**Figure S2:** Protein domain organization and lineages‐specific substitutions in SIRT1, SIK3, NFIL3, and PTBP1. Schematic diagram showing the domain boundaries and positions of lineages‐specific substitutions in four circadian‐related proteins. Functional domains are indicated by colored boxes with corresponding amino acid ranges. Vertical ticks above each protein bar mark the locations of lineages‐specific substitutions. Domain boundaries were annotated according to the UniProt database.
**Figure S3:** Sleep latency in zebrafish larvae overexpressing mouse or dolphin CSNK1E. Sleep latency was measured in zebrafish larvae overexpressing pCS2‐mCherry empty vector (control), mouse CSNK1E, or dolphin CSNK1E under a 14:10 light–dark (LD) cycle. Data are presented as mean ± SEM No significant differences in sleep latency were observed among the groups (*p* > 0.05).
**Figure S4:** Full‐length western blots correspond to PER2 stability assays. Full‐length western blot images showing PER2, CSNK1E, and GAPDH detection in HEK293T cells expressing mouse or dolphin CSNK1E after cycloheximide (CHX, 200 μg/mL) treatment for 0, 3, 6, and 9 h. Molecular weight markers and individual lanes are indicated. GAPDH was used as the loading control.

**Table S1:** Rapidly evolving genes on the terminal of 71 mammals detected by branch model.
**Table S2:** Rapidly evolving genes in the ancestor of different mammalian lineages detected by branch model.
**Table S3:** Positively selected genes and sites on the terminal of 71 mammals detected by branch‐site model.
**Table S4:** Summary of gene‐wide positive selection analysis in 20 genes using the BUSTED method implemented in Datamonkey webserver.
**Table S5:** Positively selected genes and sites in the ancestor of mammals detected by branch‐site model.
**Table S6:** Functional effect of specific amino acid replacements in 20 PER2 regulated genes among major mammalian orders.
**Table S7:** Amino acid substitutions in PER2 among polar and hibernating mammals.
**Table S8:** Functional effect of cetacean‐specific amino acid replacements.
**Table S9:** Structural alignments for circadian clock proteins from representative species.
**Table S10:** Molecular docking results.
**Table S11:** The 20 candidate genes and their protein products.
**Table S12:** Accession numbers of species in database.
**Table S13:** List of primers used to construct plasmids in this study.

## Data Availability

All data were included in the manuscript and Supporting Informations for readers to validate.
