# Supplementary material for: Selection on PER2 Phosphorylation Regulation Underlies Atypical Mammalian Circadian Rhythms
Source: Ecol Evol. 2026 Oct 15;16(10):e74524. doi: 10.1002/ece3.74524 (PMC13671881; doi:10.1002/ece3.74524)
Supplement: Supplementary file 1 [file ECE3-16-e74524-s002.docx]

**Figure S1.** **Distribution of lineage-specific amino acid substitutions in PER2 regulatory genes across mammalian circadian phenotype groups.** Horizontal bar plots show the number of PER2 regulatory genes containing lineage-specific amino acid substitutions and the total number of lineage-specific amino acid substitutions identified in each circadian phenotype group. Atypical circadian groups include USWS-associated, nocturnal, temperature-dependent, hibernating, and polar lineages, with canonical circadian mammals shown for comparison. Numbers within or adjacent to the bars indicate the corresponding counts of affected genes and amino acid substitutions.

**Figure S2. Protein domain organization and lineages-specific substitutions in SIRT1, SIK3, NFIL3, and PTBP1.** Schematic diagram showing the domain boundaries and positions of lineages-specific substitutions in four circadian-related proteins. Functional domains are indicated by colored boxes with corresponding amino acid ranges. Vertical ticks above each protein bar mark the locations of lineages-specific substitutions. Domain boundaries were annotated according to the UniProt database.

**Figure S3. Sleep latency in zebrafish larvae overexpressing mouse or dolphin CSNK1E.** Sleep latency was measured in zebrafish larvae overexpressing pCS2-mCherry empty vector (control), mouse CSNK1E, or dolphin CSNK1E under a 14:10 light–dark (LD)cycle. Data are presented as mean ± SEM. No significant differences in sleep latency were observed among the groups (p>0.05).

**Figure S4. Full-length western blots correspond to PER2 stability assays.** Full-length western blot images showing PER2, CSNK1E, and GAPDH detection in HEK293T cells expressing mouse or dolphin CSNK1E after cycloheximide (CHX, 200 μg/mL) treatment for 0, 3, 6, and 9 h. Molecular weight markers and individual lanes are indicated. GAPDH was used as the loading control.
